# Found

**Published:** 1869-09

**Authors:** 


### FOUND.

WE found the man the other day that knows everything, and it was truly refreshing. We have often wondered how people that know everything feel; they must have intense satisfaction in the fact, and great commisseration for other folks. Well, people who don't know anything are to be pitied. We hope that man (we mean our man—that Dentist) will be around again soon, for we have seen several of our friends recently who wish to know something, and they are just waiting for our man. Our man does not write, but he makes *oral communications, by the word of mouth.* T.

